# Behavioral Interventions to Increase Physical Activity Among Dementia Caregivers: A Narrative Review of Behavior Theory, Behavior Change Techniques, and Mechanisms of Behavior Change

**DOI:** 10.3390/geriatrics11050120

**Published:** 2026-09-03

**Authors:** Ashley M. Goodwin, Alex Makhnevich, Karina W. Davidson, Liron Sinvani, Mark J. Butler

**Affiliations:** 1 Northwell, New Hyde Park, NY 11042-1069, USA; amakhnev@northwell.edu (A.M.); ldanay@northwell.edu (L.S.); markbutler@northwell.edu (M.J.B.); 2 Institute of Health System Science, Feinstein Institutes for Medical Research, Manhasset, NY 11030-3816, USA; 3 Institute for Healthy Aging, Northwell, New Hyde Park, NY 11042-1069, USA; 4 Donald and Barbara Zucker School of Medicine at Hofstra/Northwell, Hempstead, NY 11549-0001, USA

**Keywords:** behavior change techniques, mechanisms of behavior change, physical activity, caregiving, dementia

## Abstract

**Background:** Due to the rise in Alzheimer disease (AD) and AD-related dementias (AD/ADRD) in an aging population, millions of caregivers will be at risk for physical and mental health decline. As those they care for already have physical and cognitive impairment, it is crucial that caregivers maintain health-promoting behaviors. Because regular physical activity (PA) prevents functional loss and improves physical health and well-being, understanding how to help caregivers adopt and sustain PA despite unique time, caregiving, and self-efficacy barriers is a pressing public-health priority. Existing health-promoting interventions among dementia caregivers, including PA, have demonstrated limited success. To overcome the fragmentation and slow synthesis of behavioral science evidence, the Human Behavior-Change Project advocates theoretical grounding, systematic mapping of intervention components (e.g., behavior change techniques [BCTs]) using standardized taxonomies, and explicit specification of mechanisms of behavior change (MoBCs). **Objective:** To examine the limited success of health-promotion interventions among dementia caregivers we aimed to conduct a narrative review to synthesize evidence from interventions that evaluated PA in dementia caregivers across three domains: (1) use of behavioral theory to inform intervention design, (2) specification of BCTs, and (3) identification and measurement of MoBCs. **Methods:** Two scientific databases were searched to identify representative studies and reviews, using key terms including “physical activity”, “intervention”, “dementia”, and “caregiver” from inception to July 2025. **Results:** Seven primary intervention trials and six reviews were selected for inclusion. Gaps across the domains were identified to inform priority areas for future research. **Conclusions:** There is a need to develop tailored, theory-driven PA interventions that target empirically supported BCTs and MoBCs to improve dementia caregivers’ health and well-being.

## 1. Introduction

The US population is aging, with one in five Americans expected to be over 65 years of age by 2034, and by 2060 that ratio is expected to become one in four [1,2]. Diseases common in older adults, such as Alzheimer disease (AD) and AD-related dementias (AD/ADRD), will therefore affect large numbers of Americans. Thus, with an aging population and projected increases in AD/ADRD, the number of caregivers and caregiver burden will increase. AD/ADRD caregiving is demanding [3], with the need for care increasing based on dementia severity, neuropsychiatric symptoms and functioning [4]. This burden of providing care for persons living with AD/ADRD adversely affects caregivers’ physical and mental health [5,6,7,8], in addition to decreasing their quality of life (QoL) [9,10] and financial well-being [11,12]. Caregivers report poorer self-rated health and more days with poor physical health compared to non-caregivers [13,14], are at increased risk for chronic disease due to the stress and demands of caregiving [15], and frequently experience higher levels of depression, anxiety, and stress compared to non-caregivers [14,16]. Further, physical health issues tend to be more pronounced in caregivers dealing with dementia-related stressors [17]. Engaging in regular physical activity (PA) is one way caregivers can combat some of this burden.

Regular PA is a well-established strategy to improve physical and mental health, with all major PA guidelines recommending 150 min/week of moderate-to-vigorous PA [18,19]. Regular PA reduces all-cause mortality [20], cardiovascular disease risk [19], and improves physical functioning and mental health [18,19,21]. For caregivers, increased PA improves their physical health and well-being [22], particularly in enhancing their psychological well-being (stress, depression) and QoL [23,24,25,26]. Caregivers who engage in PA programs such as brisk walking report higher readiness for exercise at program completion [24,27], signaling a likelihood of PA adoption over a longer term. Participation in PA can also increase self-efficacy for caregiving [24]. Yet, 60% of dementia caregivers do not meet recommended PA levels, compared with 29% of U.S. adults overall, highlighting markedly greater inactivity among caregivers [28,29].

Evidence has demonstrated that even small increases in activity, such as increasing daily steps, can improve health outcomes [20,30,31]. As little as 1000 extra steps/day can lower mortality rates [32]; behavior change PA interventions focused on walking [32,33,34] report an increase of 1000 steps/day is associated with a 7% reduction in death in patient populations [35]. Walking (i.e., daily step goals) thus provides an achievable PA target, is feasible, and improves health outcomes. Despite widespread PA recommendations, clear benefits and these achievable smaller targets, many caregivers do not regularly engage in recommended PA [28].

Caregivers express a strong desire to be more active, but they face unique barriers, such as low self-efficacy for PA, lack of time and motivation, caring demands/fatigue, and inability to leave the care recipient [36]. Evidence suggests a need for behavioral interventions to facilitate PA among dementia caregivers [36], designed to target addressable caregiving-related barriers and align with the complexities and unique issues they face [22,37]. For example, developing PA programs that are flexible and can be adapted to caregivers’ schedules and care recipient’s capabilities (e.g., PA performed in and around the home; activities done with care recipients) [38,39]. Intervention trials with behavioral components incorporate behavior change techniques (BCTs). BCTs are the observable, replicable components thought to be the “active ingredients” of behavioral interventions [40,41]. BCTs are empirically supported for health behaviors [40,41,42,43], including PA [40,43], and can be combined into multi-component interventions [44,45,46]. Additionally, to understand not only whether BCT interventions work but *how* they work, investigators must specify and measure hypothesized mechanisms of behavior change (MoBCs) [40,43]. MoBCs are the basic hypothesized causal processes through which a BCT affects behavior [47]. Yet MoBCs are rarely identified and are infrequently evaluated and empirically tested [47], leaving the causal pathways linking BCTs to PA outcomes poorly understood.

To overcome the fragmentation and slow synthesis of behavioral science evidence, including health-promoting behaviors like PA, Michie and colleagues used advances in expert consensus and information science to define, classify, and interpret the existing and accelerating evidence about BCTs [41,48]. Subsequently, the Human Behavior-Change Project (HBCP) was created to answer “the big question: ‘What works, compared with what, how well, with what exposure, with what behaviors (for how long), for whom, in what settings and why?’” [49]. HBCP advocates theoretical grounding, systematic mapping of intervention components (e.g., BCTs) using standardized taxonomies, and explicit specification of MoBCs. Guided by the HBCP framework, the present narrative review synthesizes evidence from behavior change PA interventions that evaluated PA in dementia caregivers to determine which behavioral theories, BCTs and MoBCs have been used and measured in PA interventions for dementia caregivers and to identify priority gaps for future research.

## 2. Methods

We conducted a narrative review to synthesize evidence on the application of behavioral theory, behavior change techniques (BCTs), and mechanisms of behavior change (MoBCs) in physical activity interventions for dementia caregivers. Consistent with a narrative review approach to identify representative studies and reviews, we searched two scientific databases. PubMed and Google Scholar databases were searched using key terms including “physical activity”, “intervention”, “dementia”, and “caregiver”. The searches were conducted in July 2025. The articles searched were published from database inception to July 2025. Intervention trials to increase PA were prioritized while systematic reviews, meta-analyses or other reviews were considered based on relevance. We included reviews if they examined PA interventions among dementia caregivers or broader caregiver populations and provided extractable information about use of behavioral theory, BCTs, and/or MoBCs. Inclusion of reviews was thought to provide important triangulation of findings and context for interpreting the limited primary trial evidence. Only English articles with the full text available were reviewed; protocol papers, conference abstracts and thesis papers were excluded. All returned results in PubMed were screened, whereas the first 10 pages of results in Google Scholar were screened. Titles and abstracts were screened for relevancy to the aim of the review, which was to identify interventions to increase PA in dementia caregivers, and to determine the presence of behavior change interventions (i.e., behavior change theory and/or BCTs) and/or identification of and/or measurement of MoBCs. Studies that met screening criteria were fully reviewed if the primary outcome was increased PA, if change in PA was measured and reported when it was not the primary outcome, or if behavioral interventions were reviewed in the context of the effects of PA among dementia caregivers. The reference lists of selected articles were reviewed for additional studies. Selected article titles also were entered into Google Scholar and the “related articles” link was explored for any additional studies, up to the first 10 pages (i.e., up to 100 articles each), to further identify articles related to physical activity among caregivers. Finally, major publications in the AD/ADRD research area, such as the annual Alzheimer’s Association Facts and Figures, were fully reviewed for any additional studies relevant to the aim of the review.

Article selection and data extraction were conducted by a single author (AMG), who developed an initial extraction table, which was piloted by the senior author (MJB) and revised to incorporate refinements. Article content was reviewed by two of the co-authors (LS and MJB). Any uncertainties or differences in extraction were discussed and resolved to reach a consensus between these two authors. The summary tables (Table 1 and Table 2) were created once the extraction table was finalized (Supplemental Table S1). All co-authors reviewed the refined extraction table and summary tables, and interpreted and summarized the findings for integration into the manuscript.

*Quality Assessment:* Given the heterogeneity of included study designs (ranging from small feasibility trials to larger randomized controlled trials), two authors (MJB and AMG) conducted a quality appraisal of the seven primary intervention trials using key domains adapted from established tools (Joanna Briggs Institute critical appraisal tools), to create a fit-for-purpose quality appraisal. We assessed: (1) study design rigor (presence of randomization and control group); (2) statistical conclusion (sample size adequacy for power); (3) PA outcome measurement quality (objective vs. self-report); (4) participant retention (reporting of attrition/follow-up); (5) reporting completeness (intervention components and primary outcome); (6) mechanism of behavior change (identified and measured); and (7) behavioral theoretical framework (used to ground intervention development). Quality ratings (lower, moderate, higher quality) were scored based on the cumulative assessment across these domains, where ratings included “yes” (1 point), “no” (0 points), “unclear” (0 points) or “not applicable” (excluded from total question count). Points across domains were summed and divided by total applicable points to calculate a quality score: lower quality: 0–49% of criteria met; moderate quality: 50–69% of criteria met; higher quality: ≥70% of criteria met. Consensus was reached among the authors for final scoring after discussion of any discrepancies. The results are presented in Supplemental Table S2.

Throughout this review, we use “physical activity” (PA) as a term referring to any bodily movement produced by skeletal muscles that results in energy expenditure, consistent with standard definitions [50]. Exercise is by definition a form of PA, as “Exercise” refers to a structured, planned subset of PA undertaken to improve or maintain physical fitness [50]. When studies specify “moderate-to-vigorous physical activity” (MVPA), this refers to activity at ≥3 metabolic equivalents (METs) that includes both moderate-intensity (e.g., brisk walking) and vigorous-intensity activity. Where possible, we report the specific PA construct measured in each study, recognizing that interventions varied in whether they targeted general PA, MVPA specifically, structured exercise programs, or daily step counts.

**Table 1:** Summary of selected primary intervention trials (*n* = 7).

No.	First Author, Year	Country	Participants	Age (years)	N total (M/F)	Intervention *	Duration	Primary Outcome	Theoretical Framework (s)	BCT (s) **	No. BCTs Used	MoBC (s)
1	Castro et al., 2002 [51]	USA	sedentary women caring for relatives with dementia	62.73 (9.16) mean(SD)	100 (0/100)	EXP: home-based moderate-intensity endurance exercise training condition; CON: attention control (nutrition education)	12-mo	exercise retention and adherence rates to the exercise program ***	social cognitive theory	goal setting, self-monitoring, feedback, social support, restructuring the physical environment, non-specific reward, problem solving	7	none
2	Connell and Janevic, 2008/2009 [52]	USA	women caring for a spouse with dementia	66.8 (9.4) mean(SD)	137 (0/137)	EXP: telephone-based exercise program >30 min aerobic exercise 3x/week plus stretching and strength training; CON: written materials about PA	12-mo	time spent in aerobic exercise 6 months and 12 months from baseline	social cognitive theory; motivational interviewing	goal setting, feedback, demonstration of behavior, instruction on how to perform a behavior, self-monitoring, problem solving, verbal persuasion about capability	7	exercise self-efficacy; self-efficacy for self-care
3	Dios-Rodríguez et al., 2023 [53]	Spain	PLWD and their caregivers (dyads)	PLWD 82 (median); CG 62 (median)	140 PLWD (51/89); 176 CG (48/128)	EXP: 30 min MPA 5 days/week or 20 min VPA 3 days/week; CON: normal care and delayed any systematic intervention in PA	6-mo	change in MPA and VPA from baseline to 6 months	none	strategies not codable to BCTs (telephone interviews: various strategies offering support for PA)	0	none
4	Farran et al., 2008 [54]	USA	caregivers of persons with AD	65.4 (10.8) mean(SD)	15 (7/8)	Multicomponent health promotion [endurance, strength/balance, and flexibility to engage in MPA 30 min/day most days of the week for a total of >150 min/week]; CON: None	6-mo	mean change in PA behavior	self-efficacy theory; social learning theory	goal setting, self-monitoring, feedback, problem solving, action planning	5	none
5	Farran et al., 2016 [55]	USA	caregivers of persons with AD or related dementia	61 (12) mean(SD)	211 (38/173)	EXP: Enhancing Physical Activity Intervention (EPAI) to accrue ≥ 150 min/week MVPA; CON: Caregiver Skill Building Intervention (CSBI) control	12-mo	minutes spent in MVPA	stress process model; social cognitive interaction model of health behavior	goal setting, feedback, behavioral rehearsal, self-monitoring, problem solving, environmental restructuring, prompts/cues, verbal persuasion about capability	8	self-efficacy
6	Hirano et al., 2011 [56]	Japan	caregivers living with older patients with AD	73.7 (4.4) mean (SD)	31 (10/21)	EXP: home-based moderate intensity exercise 3x/week; CON: did not receive exercise prescription	3-mo	change in level of daily PA	none	none	0	none
7	Ptomey et al., 2019 [57]	USA	persons with AD and their caregiver (dyads)	AD: 74.1 (10.2); CG: 67 (13.0) mean(SD)	9 dyads (AD 4/5; CG 6/3)	EXP: home-based 30-min group exercise sessions 3x/wk via video conferencing; CON: None	3-mo	feasibility of group video conference approach for increasing MPA ***	none	self-monitoring, goal setting, social support, behavioral practice/rehearsal	4	none

BCT: behavior change technique; MoBC: mechanism of behavior change; EXP: experimental condition; CON: control condition; PA: physical activity; MPA: moderate physical activity; VPA: vigorous physical activity; MVPA: moderate-to-vigorous physical activity; PLWD: persons living with dementia; AD: Alzheimer disease. Notes: * Additional details of interventions and results are available in Supplemental Table S3. ** BCTs were coded to the BCTTv1 taxonomy (Michie et al., 2013 [41]) and are reported as the short label from the taxonomy. Where intervention descriptions lacked sufficient detail to confidently map to a BCTTv1 label, we report the component textually and annotate it as “uncodable.” *** Physical activity not the primary outcome but measured as a secondary outcome.

**Table 2:** Summary of selected reviews (n = 6).

Type	No.	First Author, Year	Country	Participants	Age (yr)	No Studies/N Total	Theoretical Framework (s)	BCT (s)	MoBC (s)
Reviews of PA Interventions Among Dementia Caregivers	1	Baik et al., 2021 [58]	USA, India, Australia, Brazil, Canada, Japan, Turkey	Family caregivers (Alzheimer or dementia [n = 7], cancer [n = 4], stroke [n = 2], heart failure [n = 1], neurological rehab [n = 1], or psychosis [n = 1])	mean range 48 to 74	16/918	stress process model; social cognitive interaction model of health behavior	goal setting, feedback, behavioral rehearsal, self-monitoring, problem solving, restructuring the environment, prompts/cues, self-belief	self-efficacy
	2	Lambert et al., 2016 [24]	USA, Brazil, Australia, Sweden, Canada, India, Japan	Family/informal caregivers (Alzheimer’s/dementia [n = 9]; stroke [n = 1], cancer [n = 1], psychosis [n = 1], and other [n = 2])	mean range 41–73.7	14/693	social cognitive theory; motivational interviewing; self-efficacy theory; social learning theory	goal setting, feedback on behavior, demonstration of behavior, shaping knowledge, self-monitoring; problem solving, self-belief	exercise self-efficacy; self-efficacy for self-care; self-efficacy for PA
	3	Teri et al., 2020 [59]	USA	Community-dwelling persons with dementia (PWDs) and their family caregivers (dyads)	PWD mean 81.3; caregiver mean 68.7	10/255 (dyads)	behavioral and gerontological theory	goal setting; problem solving; social support; strategies not codable to BCTs: identifying and decreasing triggers and reinforcers of negative behaviors; opportunities and encouragement for positive, pleasant activities and interactions	None
Reviews of non-PA Behavioral Interventions Among Dementia Caregivers	4	Ahn et al., 2025 [60]	China, Japan, Spain, UK, USA	Informal caregivers of people with Alzheimer’s disease and related dementias	Mean 64 range 57.0–73.7	12/1163	social cognitive theory; motivational interviewing	goal setting; feedback; behavioral rehearsal; self-monitoring; problem solving; restructuring the environment; prompts/cues; self-belief; social support; non-specific reward; shaping knowledge	exercise self-efficacy; self-efficacy for self-care; self-efficacy for PA
	5	Guay et al., 2017 [61]	USA, France, Netherlands, Australia, South Korea, UK, Spain, and Greece	Caregivers of people aged 50 years and older (dementia [n = 4], stroke [n = 4], cancer [n = 3], or traumatic brain injury [n = 1])	Mean(SD) range 46.9 (12.2)–64.2 (10.3)	12/1852	stress process model; stress and coping model; social learning theory; cognitive theory; self-regulation/control theory; psychoeducational intervention	social support; shaping knowledge; problem solving; reduce negative emotions; action planning; prompts/cues; demonstration of behavior; goal setting; behavioral practice; self-monitoring; feedback; credible source; social comparison; information on health consequences	intention to get support; social support; coping; self-efficacy; knowledge; skill application; perceived bonding
	6	Wu et al., 2022 [62]	USA, Europe, Canada, India, Mexico/South America	Informal caregivers of people living with dementia—spouses and adult children	Mean 61.5 (SD 7.4) years (reported study means ranged 44–76 yr)	32/3866	stress and coping model; cognitive behavioral therapy; psychoeducation; transition theory; trigger behavior response; stress process model; adaption-coping model; social cognitive theory; communities of practice theory	goal setting; action planning; feedback; self-monitoring; social support; shaping knowledge; natural consequences; comparison of behaviors; associations; repetition and substitution; comparison of outcomes; reward and threat; regulation; antecedents; identity; scheduled consequences; self-belief; covert learning	caregiver burden/strain; self-efficacy/confidence; caregiver stress/distress; social support; caregiving competence/skill mastery; stress reactivity; self-regulation; interpersonal

Additional details of review characteristics and results are available in Supplemental Table S4.

## 3. Results

### 3.1. Search Results

A total of 361 titles were screened for review (n = 261 for PubMed and n = 100 for Google Scholar). Of these, n = 49 were selected for full review (n = 21 from PubMed and n = 28 from Google Scholar). A compilation of the reviewed articles is available in Supplementary Table S1, comprising 28 intervention trials and 21 reviews (including n = 5 duplicates). After duplicates were removed, n = 44 were fully reviewed, and of these n = 13 were selected to be included in the review based on relevancy to the aims (i.e., 31 were excluded). N = 7 were PA intervention trials among caregivers and n = 6 were reviews of the effects of interventions among caregivers on physical and mental health outcomes. Of the 31 excluded articles, reasons for exclusion primarily included no measurement of change in PA (n = 24, 77%). Three of the six reviews included trials that explicitly measured change in PA behavior in caregivers among other physical and mental health outcomes; however the other three selected reviews contained important evidence regarding behavior theory, use of BCTs, and/or MoBCs in caregiver intervention trials upon full read-through, so these were deemed important to include. Although n = 6 articles were additionally selected for review from the reference list or “related articles” search, none were included in the review. The results are organized around the three domains of interest for this review: (1) use of behavior change theory to inform intervention design; (2) specification and deployment of BCTs; and (3) hypothesized and measured mechanisms of behavior change. We first summarize evidence from the seven primary intervention trials and then synthesize corroborating findings from the six included reviews.

### 3.2. Primary Intervention Trials

The seven included primary intervention trials enrolled between nine and 211 caregivers and evaluated a range of home-based, telephone-supported, group, and technology-delivered programs (caregiver Ns: Castro [51] N = 100; Connell & Janevic [52] N = 137; Farran 2008 [54] N = 15; Farran 2016 [55] N = 211; Hirano [56] N = 31; Ptomey [57] N = 9 dyads; Dios Rodríguez [53] N = 176 caregivers [176 caregivers; 140 persons living with dementia (dyads)]). PA interventions varied in duration, mode and type of PA prescribed. Intervention durations ranged from 3 to 12 months, and delivery modes included telephone or mail counseling, in-house visits, group sessions, and one feasibility trial using tablet/Zoom. Five studies used moderate-intensity aerobic exercise (walking or combined aerobic, strength, and balance activities) targeting ≥150 min/week or ≥30 min/session on most days [51,52,53,54,55]. Two studies prescribed structured home-based programs with counseling [51,54]; three employed telephone- or self-directed protocols [52,53,56]; and one used remote video-conferencing group sessions [55]. Farran et al. (2008) [54] was a multi-component health-promotion program incorporating endurance, strength, balance, and flexibility, with objectively measured physical activity via accelerometry but no randomized control group. In terms of efficacy for these behavioral interventions to increase PA, only two of the seven (29%) studies resulted in significant changes in PA in dementia caregivers. Connell & Janevic [52] demonstrated greater exercise increases at 6 months that were maintained at 12 months among participants with low baseline activity and also reported greater reductions in perceived stress and increases in exercise self-efficacy versus control, while Farran et al. (2016) [55] found that their Enhancing Physical Activity Intervention (EPAI) significantly increased moderate-to-vigorous PA and step counts relative to the control condition. Given the small number of trials (n = 7), heterogeneous designs, and variable sample sizes (N = 9 to N = 211), evidence for intervention efficacy remains limited. Table 1 provides details for all primary studies selected for inclusion.

*Behavioral Theory:* Of the seven intervention trials to increase PA in dementia caregivers (N = 5), or that measured changes in PA if the primary outcome was not PA (N = 2) [51,52,53,54,55,56,57], four out of seven (57%) were based in a theoretical framework [51,52,53,57]. Theoretical frameworks included social cognitive theory (N = 2) [51,52], social learning theory (N = 1) [57], and self-efficacy theory (N = 1) [57], and Farran et al. (2016) was based on two theories: the stress process model and social cognitive interaction model [55]. The two trials that demonstrated statistically significant increases in caregiver PA were both explicitly theory-driven [52,53].

*BCTs:* Five out of seven (71%) trials utilized BCTs or components that could be coded to BCTs (examples include goal setting [BCTTv1 1.1], self-monitoring [BCTTv1 2.3], feedback [BCTTv1 2.2], and problem solving [BCTTv1 1.2]) [51,52,53,55,57]. A total of 16 BCTs were identified as deployed across the five trials. The most common BCTs were goal setting (five trials) [51,52,53,55,57], self-monitoring (five trials) [51,52,53,55,57], feedback (four trials) [51,52,53,57], and problem solving (four trials) [51,52,53,57]. Among trials that reported BCT content the number of BCTs per intervention averaged approximately six (mean = 6.2; range 4–8); two studies did not use BCTs [54,56].

*MoBCs:* Only two out of the seven (29%) trials identified MoBCs [52,53]. MoBCs included self-efficacy, exercise self-efficacy, and self-efficacy for self-care. Only one trial measured change in the MoBCs (exercise self-efficacy and self-efficacy for self-care) [52]. Two of the trials that did not identify MoBCs were grounded in behavioral theory [51,57], while the remaining three also lacked explicitly citing a behavioral theory [54,55,56].

*Quality assessment of primary trials:* Quality appraisal revealed trial quality heterogeneity (Supplemental Table S2). Four of the seven trials scored as “higher quality” by meeting >70% of the pre-defined criteria, whereas two were scored as “moderate quality” (50–69% criteria met) and one was scored as “lower quality” (<50% criteria met). The two trials that resulted in significant changes in PA among dementia caregivers were both rated as “higher quality”, including theoretical grounding, study rigor, and reporting completeness.

### 3.3. Reviews

Table 2 includes details on the selected reviews. The six included reviews varied in scope. Three of the six reviews included trials that measured change in PA behavior in caregivers among other physical and mental health outcomes; however the other three selected reviews did not report changes in PA behavior but contained important evidence regarding behavior theory, use of BCTs, and/or MoBCs in caregiver intervention trials upon full read-through, so these were deemed important to include in the scope of this narrative review. Of the three reviews that examined PA interventions in caregivers and where measurement of PA was available [24,58,59], evidence suggests that increasing caregiver PA is possible, but considerable gaps remain. Each of these three reviews are now described, and the remaining three relevant reviews are described in a subsequent sub-section.

#### 3.3.1. Reviews Examining PA Interventions Among Caregivers That Included PA Measurement

Baik et al. [58] reviewed PA randomized controlled trials (RCTs) for family caregivers across chronic conditions including dementia (44% of included trials) (16 included trials; summed participants = 918). The aim of this review was to analyze and capture the most recent trends in physical activity interventions for family caregivers of older adults with chronic disease as found in randomized clinical trials over the last 10 years (2010–2020). PA level was measured in 6/16 trials included in the review as an outcome of the intervention; half of these included trials (n = 3) were in dementia caregivers. Physical activity programs with mixed modes (e.g., aerobic and resistance exercise), mixed delivery methods (e.g., in-person and telephone) and mixed settings (e.g., supervised gym-based sessions and unsupervised home-based sessions) were used across the 16 included trials. Most trials that measured PA (n = 5/6) reported significant effects of PA interventions on caregivers’ engagement in overall PA, three of which were among dementia caregivers (i.e., all included trials in dementia caregivers that measured PA levels demonstrated significant improvements in PA levels). PA interventions included in the review overall significantly improved psychological health but had inconsistent effects on physical health. The authors advocate for using digital health technology in future trials to help motivate caregivers to exercise and self-monitor their PA levels; thus, developing and testing technology-supported PA programs to determine optimal PA content and delivery methods, and to quantify caregivers’ PA in the home environment, are needed.

Lambert et al. [24] conducted a descriptive systematic review of trials examining the effects of PA interventions on caregiver and care recipient physical and mental health outcomes (14 included trials; summed participants = 693), including the majority among Alzheimer’s/dementia caregivers (n = 9/14 included trials, 64%). The aim of this review was to examine the efficacy of PA interventions on caregivers’ psychosocial outcomes (depression, stress, anxiety, distress, well-being, quality of life, anger control), burden, PA levels, physical health/functional fitness (strength, balance, gait, blood pressure, heart rate, body mass index), sleep quality, social functioning, and coping/readiness/self-efficacy, and—when reported—care recipients’ psychosocial outcomes. Six of the 14 studies measured PA as an outcome, four of which were among dementia caregivers. A variety of PA modalities and delivery formats were represented across the 6/14 included trials—walking programs (home-based, telephone-supported), yoga (group and home practice), tai chi, aerobic exercise, strength training, lifestyle PA (gardening, housework); group-based, home-based, telephone-supported, and some dyadic. Five of the six included trials reported a significant increase in caregivers’ PA, four of which were among dementia caregivers. PA interventions showed promise for other health-related outcomes: studies reported improvements in distress, well-being, QoL, sleep quality, and self-efficacy or readiness for exercise. Effects on caregiver burden and physical health were mixed (fewer than half the studies showed benefits). Attrition was lower in home-based PA interventions compared to group-based; all four studies with significant findings among dementia caregivers were home-based. The authors conclude that while most studies showed that caregivers *can* increase their PA levels, more work is needed to build stronger evidence, including using objective measures of PA, fitness, and functional outcomes, and developing dyadic PA interventions to test the effects on both the caregivers’ and patients’ reported health outcomes.

Teri et al. [59] sought to review the results across a multi-site (N = 10) translational study of an evidence-based multi-component exercise plus behavioral/psychosocial intervention to improve the health and well-being of persons with dementia (PWDs) and their family caregivers, Reducing Disability in Alzheimer’s Disease (RDAD). The 10 sites enrolled N = 255 dyads. The aim of the review was to describe the translation, implementation, and effectiveness of RDAD-NorthWest conducted by staff in regional Area Agencies on Aging (AAA). All 10 sites measured change in PA. The multi-component dyadic intervention comprised progressive aerobic/endurance (target ≥ 30 min moderate daily), lower-body strength training, balance/flexibility exercises, and caregiver training in dementia education (A-B-C problem solving, pleasant events scheduling, behavior management) delivered by trained AAA case managers with manuals, home visits, homework and fidelity monitoring. Both PWDs and caregivers significantly increased their physical activity levels during the 4-month treatment period; however only PWD PA increase remained significant at follow-up; caregiver PA levels were still higher compared to baseline. Each member of the dyad experienced other benefits during the active treatment period, including improved quality of life for PWDs and decreased depressive symptoms for caregivers; the study demonstrated feasibility of community delivery. The authors note that while caregiver/PWD gains were maintained at follow-up, it is unlikely these gains will be maintained without ongoing support from communities.

#### 3.3.2. Reviews Examining PA Interventions Among Caregivers That Did Not Include PA Measurement

We now discuss the three reviews that did not specifically examine PA behavior change in dementia caregivers but reviewed behavior change components, the use of behavior change techniques and how they impact on the efficacy of interventions on other health-related outcomes in dementia caregivers [60,61,62]. Among these three reviews evidence suggests that more theory-based interventions need to be developed in this population, with isolation of the specific effect of behavioral components and explicit reporting of behavior theory, BCTs, and MoBCs.

Ahn et al. [60] examined the effects of a PA interventions on physical health (e.g., aerobic endurance, balance, and/or strength), psychological health (e.g., anxiety, caregiver burden, depressive symptoms, and/or stress), and well-being (e.g., quality of sleep, psychological well-being, and/or health-related QoL) among informal caregivers of people with cognitive decline (12 trials included; summed participants = 1163) [60]. Four of the 12 included trials measured change in PA, but this was not reported in the review; these data were identified because these four trials are included in the primary intervention studies discussed in the previous section of this review [51,52,53,54]. Interventions across the 12 trials reviewed included walking, tai chi, or multi-component activities (aerobic, strengthening, stretching, and/or balance activities). The authors reported moderate improvements only in one physical outcome (static balance) and one well-being outcome (psychological well-being).

Guay et al. [61] examined the efficacy of internet-based interventions for caregivers of older adults, including those with dementia, based on intervention components (e.g., content, multimedia use, interactive online activities, and provision of support), behavior change techniques, and caregiver outcomes (e.g., effects on stressors, mediators, and psychological health) (12 trials included; summed participants = 1852). Interventions included in the review varied and included web-based education interventions, self-help web-based therapeutic interventions, and human-supported web-based therapeutic interventions, and used written text and/or videos, interactive online activities (homework, quizzes, and exercises) or questionnaires, and human support provided asynchronously and synchronously. Seven of the 12 (58%) interventions included in the review led to statistically significant improvements in caregiver outcomes, three of which were among dementia caregivers. The efficacious interventions used interactive components including online exercises and homework (N = 4) or questionnaires on health status (N = 2), and (N = 5) incorporated remote human support, either by professionals or peers. The authors conclude that the design and aim of the included trials did not permit determining exactly which component and/or behavior change technique was more efficacious in producing positive health outcomes in caregivers.

Wu et al. [62] mapped behavior change theories, BCT clusters, and behavior change agents (i.e., mechanisms of behavior change) involved in the web-based interventions for informal caregivers of people living with dementia; examined the relationship between behavioral change elements and retention in the studies; and examined the relationship between participant characteristics and study retention (32 trials included; summed participants = 3866). Caregiver outcomes included: caregiver health and well-being (burden, stress, depression, pain, loneliness) (25/32, 78%), perceived competence (11/32, 34%), problematic behaviors of care recipients (7/32, 22%), self-efficacy (7/32, 22%), perceived social support (6/32, 19%), quality of life (5/32, 16%), caregiving knowledge (4/32, 13%), quality of the relationship with care recipients (4/32, 13%), perceived health (4/32, 13%), and intervention usability and feasibility (4/32, 13%). Interventions included in the review examining this diverse list of caregiver health outcomes included RCTs, pilot studies, feasibility studies, mixed methods studies, and quasi-experimental studies, and most (80%) were web-based interventions only, while 20% integrated web-based intervention with other telehealth modalities (e.g., telephone, virtual reality, email contact, and video conferences). The authors advocate for explicitly reporting behavioral theory, BCTs, and mechanisms, and tailoring interventions to participant characteristics.

*Behavioral Theory:* Explicit examination of behavior change theory use was limited and, where reported, the extent of theory use varied. Ahn et al. (2025) [60] reported limited use of behavioral theory in included trials; only three of the 12 (25%) trials stated a theoretical framework (social cognitive theory (3) and motivational interviewing (1)). Similarly, Wu et al. [62] found that 53% (17/32) of web-based caregiver interventions specified a theoretical basis. Guay et al. [61] noted that interventions that led to significant caregiver outcomes typically included behavior theory. Of the six interventions that measured PA reviewed by Baik et al. [58], three were among dementia caregivers, and of these, only two specified a theoretical framework. Three out of five interventions included in the review by Lambert et al. [24] that demonstrated significant increases in PA, all among dementia caregivers, used behavior theory. Lastly, the design of RDAD-NW trials reviewed by Teri et al. [59] included a behavioral framework.

*BCTs:* All three reviews that examined intervention components in depth [58,61,62] report that BCTs are commonly used across caregiver interventions, though reporting quality varies. Guay et al. [61] identified interactive online components and professional/peer support as common elements among effective programs; the most frequently used BCTs included in efficacious interventions were provision of social support and the combination of instructions to guide behavior change and barrier identification. Ten of the 12 interventions reviewed used BCTs, seven of which (58%) led to statistically significant improvements in caregiver outcomes, and of these three were among dementia caregivers. The authors conclude that studies isolating the specific effect of components are needed to improve understanding of the underlying mechanism of behavior change. In Wu et al. [62], all 32 trials included in the review used at least one BCT, and 97% (31/32) included at least two BCTs in their intervention. The total number of individual BCTs included in the trials included in the review ranged from one to 14, with a median of five techniques and the total number of BCT clusters ranged from one to nine with a median of 4.5 clusters. Higher BCT cluster counts were associated with lower retention. Of the six included trials that measured PA reviewed by Baik et al. [58], three were among dementia caregivers. Most included trials (N = 5) reported significant effects of PA interventions on caregivers’ engagement in overall PA, including all three among dementia caregivers; of these two used BCTs. In Lambert et al. [24], three out of five interventions included in the review that demonstrated significant increases in PA, all among dementia caregivers, used at least one BCT. The design of RDAD by Teri et al. [59] included the utilization of BCTs to support caregivers. Ahn et al. [60] reported limited use of BCTs in included trials.

The reviews also highlight a practical concern, that higher BCT cluster counts were associated with lower retention in Wu et al.’s mapping exercise, and several authors caution that multi-component complexity may reduce adherence. Guay et al. [61] and Baik et al. [58] emphasize that BCTs such as social support, instruction, problem solving, goal setting, self-monitoring, and feedback are common to the more effective programs, and they recommend clearer, standardized reporting of BCT content (e.g., using BCTTv1) [40] to enable replication and synthesis.

*MoBCs:* Across the reviews, it was consistently observed that mechanisms were under-reported and under-tested. Wu et al. [62] indicated that although many included trials described behavior change agents (81% noted some agent), formal mediation testing was uncommon. Guay et al. [61] and Ahn et al. [60] similarly call attention to sparse measurement of pathways linking intervention components to outcomes. Ahn et al. noted that only one of the included PA trials they reviewed tested theoretical mechanisms [60]. Of the six included trials that measured PA reviewed by Baik et al. [58], three were among dementia caregivers, and of these, only two identified a MoBC. Among trials included in Lambert et al. [24] only one included a MoBC. The RDAD trials (N = 10) reported by Teri et al. [59] did not include MoBCs. Together, this indicates that while interventions commonly use BCTs and sometimes state theoretical rationales, few explicitly identify and empirically test the MoBCs that would explain how those techniques produce behavior change. Wu et al.’s finding that many studies include multiple BCTs but rarely test mechanisms underscores the challenge: without measurement and mediation analyses, the causal chain (BCT → MoBC → behavior change) remains largely unverified.

Cross-reference observations from reviews: Evidence that dementia caregiver PA can be increased exists but is concentrated in a subset of reviews that included trials that measured PA directly and used behavior change components [24,58,59]. Reviews converge on a recommendation to (a) improve theory specification, (b) map and report BCTs using standardized taxonomies, and (c) measure hypothesized mechanisms and use objective PA measurement (accelerometry/digital monitoring) when feasible. Authors emphasize feasibility of flexible delivery (home-based, telephone, web) often supplemented with human support, but also warn that multi-component complexity may harm retention and interpretability [62].

Table 2 provides details for all reviews selected for inclusion.

## 4. Discussion

PA promotion is inherently a behavior change challenge; therefore grounding in behavioral theory, identification of BCTs, and putative MoBCs to achieve a positive change in PA behavior is important [54]. This was the impetus for this narrative review. We found that few studies have examined the utility of behavioral intervention components for increasing PA in dementia caregivers, with only seven primary intervention studies being selected for review, four of which were guided by behavioral theory, five utilized BCTs, two identified MoBCs (only one measured change in MoBCs), and only two resulted in significant changes in PA. Similarly, in reviews of trials to improve other health and well-being domains of dementia caregivers, the lack of behavioral theory grounding the work and lack of behavior change content for caregivers contributed to their limited success. Even when BCTs were included, they were bundled into multi-BCT interventions, disallowing the unique effects of each single BCT to be elucidated. Finally, putative MoBCs were infrequently included in study designs, and even when they were, their effects were most often not measured. Collectively, this suggests a gap in the literature across these three issues. We provide a summary of each issue, in the context of the extant behavior change literature.

### 4.1. Behavioral Theory

Although theory was present in just over half of the primary trials (4/7), it is notable—albeit based on very limited evidence (n = 2 trials)—that both trials achieving significant PA increases employed explicit theoretical frameworks [52,53]. While this pattern is consistent with the broader behavior change literature showing theory-driven designs yield larger effects, the small number of efficacious trials precludes definitive conclusions. Still, this is in alignment with reporting by the included reviews, where grounding in behavioral theory was limited and studies with theory-driven designs could be linked to larger or more consistent effects [24,58,59,60,61,62]. Other reviews also corroborate that theory use is associated with larger behavioral effects [62,63]. The practical implication of these findings is that future caregiver PA interventions, including among dementia caregivers, should adopt behavioral theory guiding their design so that intervention components (i.e., BCTs) can be selected and specified in a way that targets empirically plausible pathways. social cognitive theory (SCT) [64] and self-efficacy frameworks [65], for example, recur in the caregiver PA and broader PA literature and are pragmatic candidates because they map well onto commonly used BCTs (e.g., goal setting, self-monitoring, feedback, social support) [41] and to caregiver-relevant determinants such as confidence. Explicit theory use also clarifies why particular BCTs were selected (not simply because they are common), and enables a priori tests of a key MoBC (e.g., does change in self-efficacy mediate PA change?), providing a testable causal chain (BCT → MoBC → behavior) that can be empirically evaluated, which is essential to move from promising packages to parsimonious, scalable interventions [66]. Other theories to consider depending on the intervention aim include implementation intention/planning models (for translating intentions into action) [67], Habit Formation and Automaticity frameworks (for long-term behavior change/maintenance) [68,69], and the stress process model (to situate PA within the caregiving stress context) [70,71]. Selecting a primary theory, and, if justified, a secondary complementary framework, allows targeted BCT selection and focused MoBC measurement.

### 4.2. BCT Components

BCTs are widely used to promote health behaviors, including PA, in the general population and in older adults [43,72]. However, in caregivers, our review suggests that the absence of behavior change content and use of very few or no BCTs to support caregivers in achieving better health is attributed to the lack of significant findings on caregiver PA levels and other health outcomes, in alignment with other evidence [61]. In a 2010 review of interventions to promote healthy living habits, authors found trials that were theoretically grounded and used BCTs were associated with larger effect sizes when compared with another intervention or no intervention at all [63]. Despite this evidence, a later systematic review (2017) of interventions in caregivers found that the design and aim of the included studies did not permit determining exactly which component and/or BCT was more efficacious in producing positive outcomes in caregivers [61]. Further, all but two disallowed isolation of components to assess efficacy, permitting authors to only hypothesize which combination of components and factors led to better outcomes. Three interventions that used less than two BCTS, compared to the others that used four to 10 BCTS, had no significant effects on any caregiver outcomes; this result was also reported in a review specifically for caregivers of people with dementia [73]. The authors attribute this to lack of BCT use, in alignment with our findings. These findings together suggest that assessing the unique effects of single BCTs, alone and in combination, has not been done in prior trials for caregivers of persons with dementia. Addressing caregiving-related needs related to PA participation through flexible, tailored (e.g., dementia-friendly) BCT programs may help caregivers of persons with dementia achieve and maintain their health and well-being [36].

In the context of the extant literature, until recently the findings of many behavior change interventions, and the underlying science, were often inconsistently reported [74], limiting opportunities to synthesize findings to systematically improve behavior change interventions and build forward momentum [48]. Consequently, advances in expert consensus and information science by Michie and colleagues, and several others, sought to define, classify, and interpret the existing and accelerating evidence about BCTs and MoBCs [47]. Many behavior change trials to promote PA have been conducted in the general adult population [75,76,77,78,79,80,81,82,83]; however, in alignment with our review, most use only multiple-BCT interventions. Out of 51 PA trials that were included in a systematic review, only one featured single BCTs; of the possible 26 BCTs identified across all studies, the average per intervention was six [43]. Comparatively fewer PA trials in dementia caregivers have been conducted (this review), all of which used only multiple-BCT interventions (average of six BCTs used), except two studies that did not use BCTs at all.

Most interventions designed to change health-related behaviors include many intervention components, and have large heterogeneity in effectiveness [84,85], limiting researchers’ understanding on how the different component BCTs relate to effectiveness. The literature on PA interventions across multiple populations [77,81,82,84,86,87,88,89] shows that: (1) PA interventions vary widely in BCT components used; (2) BCTs from multiple health behavior change theories are used; (3) among behavioral interventions for dementia caregivers, the effect of single BCTs is indeterminable; (4) only few studies tested whether a specific BCT improves daily PA such as walking; and (5) comparatively fewer PA interventions used remote delivery, a promising intervention format that is more conducive to meeting unique caregiver needs (e.g., no in-person visits) and also allows for scalability. Due to the standardization of BCT taxonomies by Michie and colleagues, there is an opportunity to utilize these reliable methods for specifying BCTs and behavioral MoBCs to select appropriate behavioral intervention components [41,47,48]. The lack of PA interventions in dementia caregivers, and the inconsistent use of behavior change theory and techniques, suggests an urgent need to design behavior change interventions that will identify single BCTs, used alone or in combination, to increase PA in this vulnerable population at higher risk for inactivity. For example, Webb et al. [63] conducted a meta-analysis of internet-based behavior change interventions across adult populations (85 trials; summed participants = 43,236) and reported a small but significant overall effect (d+ ≈ 0.16), with larger effects associated with greater use of theory, more BCTs, and supplementary communication modes (e.g., SMS/phone).

### 4.3. MoBCs

Even when an intervention successfully produces behavior change, scientists rarely know why or how the intervention succeeded. MoBCs are defined as the basic hypothesized causal processes through which a BCT affects behavior [47]. Empirically testing hypothesized MoBCs is critical to understanding not only which BCTs improve PA, but how they work [46,47]. Although most investigators explicitly state the MoBC thought to underlie their successful PA intervention, rarely do they empirically test whether the MoBC changed or was even engaged [47]. This was true in our review as well, as only two of the primary trials identified a MoBC, and only one measured a MoBC. Further, MoBCs were rarely identified or measured across the trials included in the review papers we included in this review. This is in agreement with Rhodes and Pfaeffli [90] who conducted a review of mediational tests of PA behavior change and concluded that which exact BCTs and which exact MoBCs are clearly engaged and cause behavior change are rarely, if ever, tested properly. Reviewing all BCT interventions that did test for changes in a key MoBC (self-efficacy to improve PA in this case), a number of limitations were noted: (1) most interventions were multi-BCT (average of six present), prohibiting the ability to precisely identify which BCT had what size effect on the MoBC; (2) quantitative assessment of BCT use was not available, prohibiting the ability to assess *if* a BCT is used, and if so did it engage the MoBC?; and (3) none of the interventions tested the full mediation model, precluding the ability to determine whether a BCT engages the MoBC, and whether the engagement of the MoBC then increased PA. This is a notable gap in understanding what intervention components are most effective (i.e., what BCTs influence PA) and *how* these interventions influence PA (via a key MoBC). For example, in a 2019 synthesis of empirical links between BCTs and their MoBCs, 55 of 277 behavior change intervention trials targeted PA and seven specifically targeted walking [44]. Self-efficacy, or the belief about one’s capability to perform a specific behavior, is the central construct of social cognitive theory, and was the key MoBC for over 35 of the 55 PA trials, and four of the seven walking trials. Self-efficacy has been found to be integral to the initiation and maintenance of PA [32,91,92,93,94,95,96,97,98], and may be the strongest MoBC linking multi-BCT interventions with increased PA [99]. Despite this strong empirical support, only one PA trial we reviewed identified and measured self-efficacy. As such, we advocate that for a proposed behavior change intervention to increase PA, researchers should select a key MoBC associated with PA behavior change [44], such as self-efficacy, then empirically select the specific BCTs from those that are also associated with self-efficacy [44].

### 4.4. Synthesis Across Evidence

A narrative synthesis of the seven trials and six reviews reveals several convergent themes. Psychosocial outcomes were the most consistently favorable domain. Multiple trials and reviews reported reductions in depressive symptoms and perceived stress. Other trials and reviews reported improvements in QoL following PA and/or behavioral intervention programs [24,51,54,58,59,60]. However, the evidence of successful behavioral interventions to increase caregiver PA is limited. Two trials [52,53] demonstrated statistically significant increases in MVPA or steps, while other trials produced mixed or non-significant changes. Similarly, two of the reviews [24,58] reported significant improvement in PA levels for trials that measured PA as an outcome, but results were mixed in the other four reviews. Objective physical health outcomes (for example strength or balance) and caregiver burden exhibited inconsistent effects across studies, suggesting heterogeneity in how interventions influence functional outcomes. Interventions that explicitly incorporated behavior change theory, clearly specified BCT content, and used supplemental contact modes (telephone or SMS follow up) tended to yield stronger behavioral outcomes, aligning with the broader web-based behavior change literature [62,63]. Finally, substantial reporting gaps remain: many caregiver trials either lack grounding in behavioral theory, omit explicit BCT specification, and/or fail to identify and measure hypothesized MoBCs; mediation analyses are rarely conducted, which restricts understanding of causal mechanisms. Lastly, while it should be noted that some of the primary intervention trials were also included as studies in some of the reviews, their overall representation among other studies was minimal (the largest proportion was four primary trials that were included in a review of 12 studies).

This narrative review identifies a limited but encouraging evidence base for PA behavior change interventions among dementia caregivers. The most reliable benefits appear in psychosocial outcomes, with limited but encouraging evidence of increased caregiver PA in trials that were theory-driven and included identifiable BCTs [52,53]. Successful trials tended to include multiple BCTs and sustained contact, but multi-component designs prevent isolation of the specific BCTs responsible for change. Mechanism testing was infrequent; self-efficacy was the most commonly hypothesized MoBC, but it was measured in only one trial [52]. Given self-efficacy’s central role in social cognitive models and its prominence in PA behavior change, future interventions would benefit from (a) predefining a MoBC (e.g., self-efficacy), (b) selecting BCTs empirically linked to that MoBC, and (c) measuring the MoBC at multiple time points to permit formal mediation testing. To efficiently identify effective BCTs and combinations, future research should consider factorial or optimization designs (e.g., MOST) that can test single and combined BCT effects while measuring MoBCs. Pragmatic choices such as flexible home or web-based delivery, supplemental contacts to support adherence, and routine mapping and reporting of BCT content using standard taxonomies (e.g., Michie et al.’s BCTTv1) [40] will improve feasibility, reproducibility, and real world impact. Further, evidence suggests that intervention characteristics may influence the impact on behavior change such as the theoretical basis of the intervention, the behavior change techniques used, and the mode of delivery [63], all considerations for future PA behavioral trials in dementia caregivers. Overall, given substantial heterogeneity in designs and sample sizes, and with only two primary intervention trials demonstrating significant PA change, the evidence base remains insufficient for robust causal inference. Our findings should be considered hypothesis-generating, identifying directions for more rigorous future research.

### 4.5. Limitations

This review is narrative and synthesizes a small and heterogeneous evidence base. Caregiver populations were highly heterogeneous across studies (e.g., spousal vs. adult child caregivers, dementia severity, co-residence), and our small sample of trials precluded examination of potential moderators of intervention effects. Our search was limited to two databases (PubMed and Google Scholar) and did not include other relevant databases such as Scopus, Web of Science, PsycINFO, CINAHL, or Embase. This limitation, combined with the narrative rather than systematic approach, may have resulted in the omission of relevant studies. However, the consistency of themes identified across our included primary trials and the six reviews we synthesized (which themselves drew from comprehensive multi-database searches) provides confidence that we have identified the key gaps in behavioral theory application, BCT specification, and MoBC measurement. There is also the possibility of single-reviewer selection bias and potential omission of relevant studies as only one author (AMG) selected the articles to be included in the review. Several reviews included overlapping primary trials (n = 4), which may increase risk of overstating interpretation of results. We identified a maximum overlap of four primary studies among 12 studies in one review. Primary trials varied in outcome measurement (self-report vs. objective), intervention content, duration, and sample size, which limits generalizability. The included trials were also conducted predominantly in the USA and Western countries, limiting cross-cultural generalizability. Cultural factors may influence caregiver barriers, preferences for intervention delivery, and effectiveness of specific BCTs. Lastly, only English articles were considered, potentially causing publication bias.

## 5. Conclusions

An apt characterization in the field of dementia caregiver behavior change interventions is that it is rich in interventions but poor in causal mechanisms. This gap represents both a limitation of current evidence and a priority opportunity for future research. The findings should be considered hypothesis-generating rather than conclusive. There is a need for scalable, theory-driven PA behavior change interventions tailored for dementia caregivers. Priority actions are: (1) grounding intervention designs in behavioral theory; (2) transparent specification and reporting of BCTs using standardized taxonomies; and (3) routine measurement and mediation testing of hypothesized MoBCs (e.g., self-efficacy). Further, employing randomized experimental designs capable of testing single BCT and combined BCT effects (e.g., factorial/MOST designs) would improve rigor. Having a better understanding of the current gaps in the current evidence allows for the design of rigorous trials for caregivers of persons with AD/ADRD, allowing us to design successful interventions to improve the health and well-being of dementia caregivers.

## Data Availability

No new data were created or analyzed in this study. Data sharing is not applicable to this article.

## Supplementary Materials

The following supporting information can be downloaded at https://www.mdpi.com/article/10.3390/geriatrics11050120/s1, Table S1. Compilation of all articles selected for full review (extraction table); Table S2. Quality assessment of primary intervention trials; Table S3. Additional details of primary intervention trials and results; and Table S4. Additional details of review characteristics and results.
