# Supplementary material for: Behavioral Interventions to Increase Physical Activity Among Dementia Caregivers: A Narrative Review of Behavior Theory, Behavior Change Techniques, and Mechanisms of Behavior Change
Source: Geriatrics (Basel). 2026 Sep 3;11(5):120. doi: 10.3390/geriatrics11050120 (PMC13600195; doi:10.3390/geriatrics11050120)
Supplement: Supplementary file 1 [file geriatrics-11-00120-s001.zip › geriatrics-4359479-supplementary.pdf]

Table S1. Compilation of all articles selected for full review (extraction table)

No.	First Author, year	Type	Reason for Exclusion	Duplicate (Y/N)
**PubMed**				
1	Chew, 2015	Primary intervention	Change in PA behavior not measured	N
2	Cothran, 2017	Primary intervention (secondary analysis)	Secondary analysis of RCT already included (Farran et al 2016)	N
3	Dal Bello-Haas, 2014	Primary intervention	Change in PA behavior not measured	N
4	Di Lorito, 2022	Primary intervention (secondary analysis)	Caregivers not included	N
5	DiZazzo-Miller, 2017	Primary intervention	Change in PA behavior not measured	N
6	Farran, 2016	Primary intervention	Included	Y
7	Farran, 2015	Primary intervention	Same PA results reported in Farran et al 2016	Y
8	Ferrero-Sereno, 2025	Review article	Change in PA behavior not measured	N
9	Fleiner, 2020	Primary intervention	Change in PA behavior not measured	N
10	Hirano, 2011	Primary intervention	Included	N
11	Lai, 2020	Primary intervention	Change in PA behavior not measured	N
12	Lamotte, 2017	Review article	Change in PA behavior not measured	Y
13	Lang, 2023	Primary intervention	Change in PA behavior not measured	N
14	Lin, 2020	Primary intervention	Change in exergaming PA	N
15	Madruga, 2021	Primary intervention	Change in PA behavior not measured	Y
16	Mehling, 2020	Primary intervention	Change in PA behavior not measured	N
17	Nehen, 2015	Review article	Caregivers not included	N
18	Ptomey, 2020a	Primary intervention	Included	N
19	Ptomey, 2020b	Primary intervention	Change in PA behavior not measured	Y
20	Shih, 2024	Primary intervention	Change in PA behavior not measured	N
21	Teri, 2020	Review article	Included	N
**Google Scholar**				
1	Ahn, 2025	Review article	Included	N
2	Baik, 2021	Review article	Included	N
3	Boots, 2013	Review article	Change in PA behavior not measured	N
4	Cardoso, 2025	Review article	Change in PA behavior not measured	N
5	Castro, 2002	Primary intervention	Included	N
6	Connell & janevic, 2008	Primary intervention	Included	N
7	Cuthbert, 2017	Review article	Change in PA behavior not measured	N
8	Dios-Rodriguez, 2023	Primary intervention	Included	N
9	Doyle, 2020	Review article	Change in PA behavior not measured	N
10	Farran, 2008	Primary intervention	Included	N
11	Farran, 2015	Primary intervention	Same PA results reported in Farran et al 2016	Y
12	Farran, 2016	same RCT as above	Included	Y
13	Guay, 2017	Review article	Included	N
14	Kim, 2023	Primary intervention	Change in PA behavior not measured	N
15	King & Brassington, 1997	Primary intervention (feasibility RCT)	PA reported as adherence rates	N
16	Lambert, 2016	Review article	Included	N
17	Lamotte, 2017	Review article	Change in PA behavior not measured	Y
18	Leng, 2020	Review article	Change in PA behavior not measured	N
19	Madruga, 2021	Primary intervention	Change in PA behavior not measured	Y
20	Marshall, 2022	Review article	Change in PA behavior not measured	N
21	Oliveira, 2019	Review article	Only 2/7 trials included in review measured change in PA (both already included as primary trials)	N
22	Parker, 2008	Review article	Change in PA behavior not measured	N
23	Prieto-Prieto, 2022	Primary intervention	Change in PA behavior not measured	N
24	Ptomey, 2020	Primary intervention (protocol)	Change in PA behavior not measured (protocol)	Y
25	Rodriguez-Sanchez, 2014	Primary intervention (protocol)	Change in PA behavior not measured (protocol)	N
26	Sorensen, 2011	Review article	Change in PA behavior not measured	N
27	Webb, 2010	Review article	Change in PA behavior not measured	N
28	Wu, 2022	Review article	Included	N

Table S2. Quality assessment of primary intervention trials

	Item								
**Study**	**1**	**2**	**3**	**4**	**5**	**6**	**7**	**% Yes**	**Quality Rating**
Castro et el, 2002	+	-	-	+	+	+	+	71	Higher Quality
Connell and Janevic, 2008/2009	+	-	-	+	+	+	+	71	Higher Quality
Dios-Rodríguez et al, 2023	+	+	+	+	+	-	-	71	Higher Quality
Farran et al, 2008	-	+	-	+	+	-	+	57	Moderate Quality
Farran et al, 2016	+	+	-	+	+	-	+	71	Higher Quality
Hirano et el, 2011	+	-	-	+	-	-	-	29	Lower Quality
Ptomey et al, 2020	-	+	+	+	+	-	-	57	Moderate Quality

'+' Yes (1 point); '-' No (0 points); '?' Unclear (0 points); 'NA' Not Applicable (excluded from total question count)

Points across domains were summed and divided by total applicable points to calculate a quality score: Lower Quality: 0% - 49% of criteria met; Moderate Quality: 50% - 69% of criteria met; Higher Quality: ≥ 70% of criteria met

Items

(1) Study design rigor: presence of randomization and control group

(2) Statistical conclusion: sample size provides adequate power

(3) PA outcome measurement quality: objective measure of PA

(4) Participant retention: reporting of attrition/follow-up

(5) Reporting completeness: intervention components and primary outcome

(6) Mechanism of behavior change: identified and measured

(7) Behavioral theoretical framework: used to ground intervention development

Table S3. Additional details of primary intervention trials and results

No.	First Author, Year	Intervention	Duration	Physical Activity (PA) Outcome	Results
1	Castro et el, 2002	EXP: home-based moderate-intensity endurance exercise counseling to achieve at least 4 exercise sessions/week, for at least 30 min/session; CON: attention control (nutrition education)	12-mo	change in duration of moderate or vigorous intensity exercise during exercise sessions	Participants achieved 12-month average exercise adherence rate of 74% (ie, 3 exercise sessions/week) with an average of 35 min/session. At 12 months, EXP demonstrated increased knowledge of the benefits of exercise and increased motivational readiness for exercise compared with CON. Both groups significantly improved in perceived stress, burden, and depression. The duration of moderate or more vigorous intensity exercise increased over the year for EXP
2	Connell and Janevic, 2008/2009	EXP: telephone-based exercise program consisting of ≥30 min aerobic exercise 3x/week supplemented by stretching and strength training; CON: written materials about PA	12-mo	time spent in aerobic exercise 6 months and 12 months from baseline	Participants in EXP with at- or below-median exercise scores at baseline had a significantly greater increase in exercise at 6-month follow-up compared with CON. At 6 months, EXP had greater reductions in perceived stress relative to CON. EXP also reported significantly greater increases in exercise self-efficacy than caregivers in CON at both follow-up points
3	Dios-Rodríguez et al, 2023	EXP: 30 min MPA 5 days/week or 20 min VPA 3 days/week; participants were instructed to perform PA autonomously, preferably by walking around their neighborhood; CON: normal care and delayed any systematic intervention in PA	6-mo	change in MPA and VPA from baseline to 6 months	Changes observed in the PA of the PLWD and caregivers were not significant. For caregivers in EXP, at 6 months the number of aerobic steps increased to 356.91 steps while CON decreased 12.95 steps, resulting in net increases in favor of EXP of 369.86 steps, and EXP MVPA/min/week was 495.14 compared to 124.68 in CON for a net increase of 370.46 in favor of EXP
4	Farran et al, 2008	Multicomponent health promotion program including endurance, strength/balance, and flexibility to engage in 30 min/day MPA most days of the week for a total of ≥150 min/week; CON: None	6-mo	mean change in PA behavior	There were no significant improvements in self-reported PA for the total group. Fifty percent of caregivers increased total self-reported minutes of any PA and 42% increased total MVPA from pre-intervention to post-intervention; however, no caregiver engaged in vigorous PA. Activity counts were highest for sedentary activities, followed by light and moderate PA.
5	Farran et al, 2016	EXP: Enhancing Physical Activity Intervention (EPAI), a behavior-change intervention, MVPA treatment, where MVPA was defined as any leisure, ongoing, or planned PA, to accrue ≥ 150 min/ week (≥ 3 Metabolic Equivalents [METS]) and adapted to caregiver abilities; CON: Caregiver Skill Building Intervention (CSBI) control	12-mo	minutes spent in MVPA	EPAI significantly increased MVPA and number of steps. At 12 months EXP doubled their MVPA minutes/week while CON decreased their weekly MVPA minutes by 5%. MVPA most frequently reported by caregivers included brisk walking, heavy housework or gardening, or light strength training. At baseline, EXP reported having somewhat fewer MVPA min/week than CON; participants were categorized into: low= ≤ 150 min/week and high= ≥ 150 min/week MVPA where 63% of EXP compared to 37% of CON adhered to ≥150 min/week MVPA from 3–12 months
6	Hirano et el, 2011	EXP: home-based exercise prescription with moderate-intensity (3 metabolic equivalents [METs]) 3x/wk; CON: did not receive exercise prescription	3-mo	change in level of daily PA	Overall level of PA measured by the PA questionnaire score (QS) showed a significant increase in EXP compared to CON, with increases mainly explained by increase of sport activity score (SS) since no other subscales (household activity score [HS] or leisure-time activity score [LS]) showed significant changes. Significant reductions in burden and frequency of feeling fatigued, and improvement in quality of sleep in EXP at follow-up whereas no such changes observed in CON
7	Ptomey et al, 2020	EXP: home-based exercise sessions 30 min/ea delivered via video conferencing (zoom) on a tablet device 3x/week to groups of 5–8 dyads, each in their own home; CON: None	3-mo	minutes of MPA achieved during the group exercise sessions; changes in daily PA	Weekly MPA increased from the start (weeks 1–3) to end of the study (weeks 10–12) in both adults with AD (24 minutes/wk., 49%) and caregivers (49 minutes/wk., 30%); light physical activity increased in both adults with AD (36.4 minutes/wk., 11%) and caregivers (11.6 minutes/wk, 3%); no statistical tests were performed to detect significance of increases in PA because the study was powered on feasibility as the primary outcome.

PA: physical activity; EXP: experimental condition; CON: control condition; MPA: moderate physical activity; VPA: vigorous physical activity; MVPA: moderate-to-vigorous physical activity

Table S4. Additional details of review characteristics and results

Type	No.	First Author, Year	Objective	Duration (range)	Results
Reviews of PA Interventions Among Dementia Caregivers	1	Baik et al., 2021	To analyze and capture the most recent trends in physical activity interventions for family caregivers of older adults with chronic disease as found in randomized clinical trials over the last 10 years (2010-2020)	1 -12 months	Physical activity level was measured in 6 of the 16 studies as outcomes of the physical activity interventions; half of these (n=3) were in dementia caregivers. Most of the physical activity studies (5/6) reported significant effects of interventions on family caregivers' engagement in overall physical activity, 3/5 (60%) of which were among dementia caregivers (i.e., all studies in dementia caregivers that measured physical activity levels demonstrated significant improvements in physical activity levels). Of these, 2/5 specified a theoretical framework, used BCTs, and identified a MoBC
	2	Lambert et al., 2016	To examine the efficacy of physical activity (PA) interventions for caregivers on caregivers' psychosocial outcomes, PA levels, physical health, and—when reported—care recipients' psychosocial outcomes (n=2)	6 weeks – 12 months	Physical activity level was measured in 6 of the 14 studies as an outcome of the included interventions; 4 of which were among dementia caregivers. Five of the 6 trials that measured PA reported a significant increase in caregivers' PA, 4 of which were among dementia caregivers. Physical activity interventions significantly decreased caregivers' distress and increased their well-being, quality of life, sleep quality, physical activity levels, self-efficacy for caregiving or exercise, and readiness for exercise. Only 2 of 6 of the included studies that measured PA specified a theoretical framework, 2/6 used BCTs, and 1/6 identified a MoBC
	3	Teri et al., 2020	A review of the results of the 10 sites administering RDAD in order to translate, implement, and evaluate the effectiveness of the multicomponent RDAD program on PWD and caregiver physical activity, function, mood, quality of life, and affect	6 weeks; 13-month follow-up	Exercise days (number of days walking or other aerobic exercise for at least 30 min) increased significantly from pre- to post-treatment for both PWDs and caregivers but was only maintained at 13 months for PWD. The trials were ground in a theoretical framework and used a combination of BCTs that were able to be coded to the BCTTv1 taxonomy and BCTs that were not codable. No MoBC was identified.
Reviews of non-PA Behavioral Interventions Among Dementia Caregivers	4	Ahn et al, 2025	To systematically evaluate the effects of physical activity on physical and psychological health and well-being among informal caregivers of people with cognitive decline	8 - 48 weeks	Physical activity level was measured in 4 of the 12 studies as an outcome of the included interventions (although this was not specifically reported by the authors). Physical activity, consisting of walking, Tai Chi, or multicomponent activities (aerobic, strengthening, stretching, and/or balance activities), exerted beneficial effects on static balance and psychological well-being. Only 3 of 4 of the included studies that measured PA specified a theoretical framework, 3/4 used BCTs, and 1/4 identified a MoBC
	5	Guay et al, 2017	To provide an update on the best available evidence about the efficacy of Internet-based interventions for caregivers of older adults. Specifically, the components and the use of behavior change techniques and how they impact on the efficacy of the intervention were sought	30 days - 2 years	Internet-based interventions with professional and social support, and instructions to change behavior and problem solve in an interactive manner lead to positive outcomes in caregivers. Efficacious interventions used interactive components, such as online exercises and homework (4/12 studies) or questionnaires on health status (2/12 studies) and incorporated remote human support, either by professionals or peers (5/12 studies). The most frequently used BCTs included social support (6/12 studies) and combinations of instructions to guide behavior change and barrier identification (5/12 studies). A total of 15 BCTs were identified across studies. 58% (7/12 studies) led to significant improvement in caregiver outcomes; all of which used BCTs or behavior theory, and 4 of which were among dementia caregivers
	6	Wu et al., 2022	To map behavioral factors (BCTs [v1], behavior change theories, behavior change agents) involved in the web-based interventions for informal caregivers of people living with dementia; to examine the relationship between behavioral change elements and retention in the studies; and to examine the relationship between participant characteristics and study retention	2 weeks to 12 months	53% (17/32) of the studies used behavior change theories; 81% (26/32) included behavior change agents (aka MoBCs); stress reactivity (20/26, 77%) was the most common MoBC followed by self-regulation (18/26, 69%), and the least common was interpersonal (11/26, 42%). All 32 articles included at least one BCT, and 97% (31/32) included at least 2 BCTs; total number of individual BCTs ranged from 1 to 14, with a median of 5 (IQR 4) techniques; total number of BCTv1 clusters ranged from 1 to 9 with a median of 4.5 (IQR 3) clusters
